# Insights into the Chemical Composition and In Vitro Bioactive Properties of Mangosteen (*Garcinia mangostana* L.) Pericarp

**DOI:** 10.3390/foods12050994

**Published:** 2023-02-26

**Authors:** Bianca R. Albuquerque, Maria Inês Dias, José Pinela, Ricardo C. Calhelha, Tânia C. S. P. Pires, Maria José Alves, Rúbia C. G. Corrêa, Isabel C. F. R. Ferreira, Maria Beatriz P. P. Oliveira, Lillian Barros

**Affiliations:** 1 Centro de Investigação de Montanha (CIMO), Instituto Politécnico de Bragança, Campus de Santa Apolónia, 5300-253 Bragança, Portugal; 2 Laboratório Associado para a Sustentabilidade e Tecnologia em Regiões de Montanha (SusTEC), Instituto Politécnico de Bragança, Campus de Santa Apolónia, 5300-253 Bragança, Portugal; 3 REQUIMTE/LAQV, Department of Chemical Sciences, Faculty of Pharmacy, University of Porto, Rua Jorge Viterbo Ferreira n° 228, 4050-313 Porto, Portugal; 4 Nutrition and Bromatology Group, Department of Analytical Chemistry and Food Science, Faculty of Science, Universidade de Vigo, E32004 Ourense, Spain; 5 Programa de Pós-Graduação em Tecnologias Limpas, Instituto Cesumar de Ciência, Tecnologia e Inovação—ICETI, Universidade Cesumar—UNICESUMAR, Maringá 87050-390, PR, Brazil

**Keywords:** food waste recovery, bioactive compounds, flavonoids, antiproliferative activity, food supplement

## Abstract

The industrial processing of mangosteen (*Garcinia mangostana* L.) generates high amounts of waste, as ~60% of the fruit is formed by an inedible pericarp. However, its pericarp has been explored as a source of xanthones; nevertheless, studies addressing the recovery of other chemical compounds from such biomass are still scarce. Hence, this study intended to elucidate the chemical composition of the mangosteen pericarp, including fat-soluble (tocopherols and fatty acids) and water-soluble (organic acids and phenolic compound non-xanthones) compounds present in the following extracts: hydroethanolic (MT80), ethanolic (MTE), and aqueous (MTW). In addition, the antioxidant, anti-inflammatory, antiproliferative and antibacterial potentials of the extracts were assessed. The mangosteen pericarp showed a composition with seven organic acids, three tocopherol isomers, four fatty acids and fifteen phenolic compounds. Regarding the extraction of phenolics, the MT80 was the most efficient (54 mg/g extract), followed by MTE (19.79 mg/g extract) and MTW (4.011 mg/g extract). All extracts showed antioxidant and antibacterial activities; however, MT80 and MTE extracts were more efficient than MTW. Only MTW did not show anti-inflammatory properties, whereas MTE and MT80 showed inhibitory activities towards tumor cell lines. Notwithstanding, MTE showed cytotoxicity towards normal cells. Our findings support the idea that the ripe mangosteen pericarp is a source of bioactive compounds, although their recovery is dependent on the extraction solvent.

## 1. Introduction

The global agri-food sector generates significant volumes of food waste each year. For instance, the processing of fruit yields significant quantities of biowaste, which account for between 25 and 60% of the fruit’s weight. In turn, this biowaste is mainly composed of peels and seed that have a chemical composition rich in bioactive compounds, that can be recovered and used to create health supplements or added to food products to improve their nutritional content [1].

**Figure 1:**
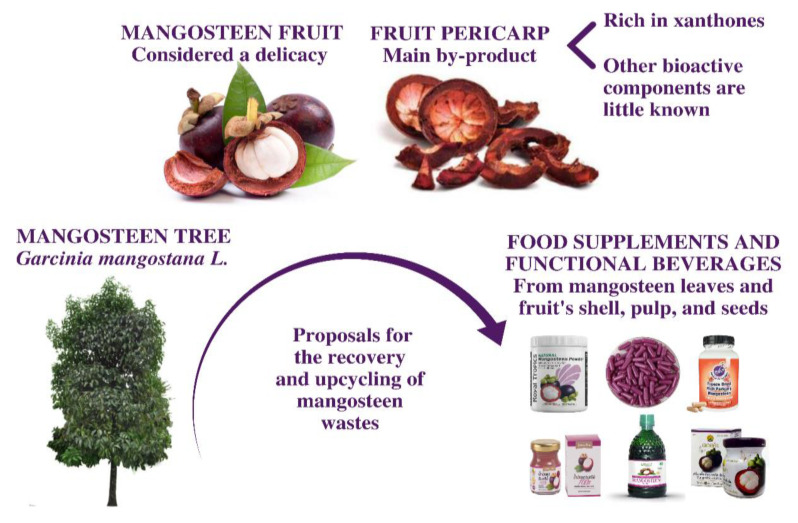
The *Garcinia mangostana* tree, its major in natura product (fruit), the major industrial by-product of its processing (fruit pericarp), and mangosteen derived-products with high added value.

*Garcinia mangostana* L. is a tropical shrub belonging to the family Clusiaceae, native to South Asia, which can also be found in other tropical territories such as South America [2]. Due to its unique form and flavor, mangosteen fruit is acknowledged as “the queen of fruit” (Figure 1). Mangosteen is a spherical fruit; when immature, it has a green color, and when entirely mature, its color is completely purple. This fruit holds many edible sweet petals (pulp), with delicious and widely appreciated unique flavor and aroma—so much that mangosteen is considered a delicacy worldwide [3].

Indeed, the edible part of the mangosteen is small, as more than 60% of the whole fruit comprises an inedible tick dark purple or reddish pericarp, which leads to a high amount of residue production after consumption/fruit processing. It is estimated that approximately 30.8 million tons of mangosteen pericarp waste are produced per year around the planet [4,5]. On the other hand, in traditional medicine, the mangosteen fruit shell has been used for the treatment of several ailments, such as skin infections, diarrhea, and fever [5]; in addition, in some regions of South America, it is employed in the preparation of digestive and energetic teas [6]. In the past few years, mangosteen fruit, including its pericarp, has been exploited for the acquisition of manifold dietary supplements, essentially capsules and functional drinks. Such products normally claim to improve the immune system, protect against free radicals, reduce allergic reactions and weight loss, among other health-promoting properties [7,8,9]. Notwithstanding, most of these potential health effects are not scientifically supported [10] for the pharmaceutical use of mangosteen. Moreover, the greater part of existing scientific evidence on mangosteen fruit and its by-products, including original articles and reviews, only addresses its xanthones compounds and corresponding bioactivities [3,11,12]. In addition, the correlation between the mangosteen chemical composition and its bioactive profile has not been completely elucidated yet [7,13,14].

Considering all of the above, this study aimed to elucidate the phytochemical profile of the mangosteen pericarp, including lipophilic compounds such as tocopherols and fatty acids, and hydrophilic compounds, namely organic acids, and phenolic compounds, including anthocyanins. Furthermore, diverse in vitro assays were performed to evaluate the antioxidant, anti-inflammatory, antiproliferative, and antibacterial potential of the hydroethanolic (MT80), ethanolic (MTE) and aqueous (MTW) extracts obtained from this by-product.

## 2. Materials and Methods

### 2.1. Fruit Material

Ripe mangosteen fruits with completely purple pericarp were acquired locally in Bragança, Portugal. Fruits were washed in current water and the pericarp and pulp were manually separated. Then, the pericarps were frozen (−20 °C), lyophilized, and ground into uniform particles, which were kept frozen until analysis.

### 2.2. Assessment of Chemical Composition

#### 2.2.1. Organic Acids

Organic acids were recovered from the sample (1 g) by maceration (room temperature for 20 min) with 25 mL metaphosphoric acid (4.5% (*w*/*v*)). The extract obtained was injected in a Shimadzu 20A series UFLC-PDA. A C_18_ column was used to separate the compounds, and sulphuric acid (3.6 mM) was used for the elution, with a flow rate of 0.8 mL/min. The preferred wavelengths for detection in a PDA were 215 and 245 nm (for ascorbic acid). Compounds were identified by comparing the area of extract peaks with calibration curves produced from commercial standards. Data were presented in mg per 100 g of dry pericarp (dw).

#### 2.2.2. Tocopherols

Tocopherols were extracted from the sample (500 mg) through successive homogenization and centrifugation (4000× *g* at 10 °C for 5 min, three times) with methanol and hexane, and the supernatant phase was gathered, dried in a flow of nitrogen, re-diluted in hexane (2 mL), and analyzed using a HPLC-FL [15]. For quantification, genuine standards of α-, β-, γ-, and δ-tocopherol, as well as tocol (internal standard) were used. The results were reported in mg per 100 g of dw.

#### 2.2.3. Fatty Acids

Fatty acids were obtained from the lipid fraction assisted by Soxhlet extraction, followed by methylation with 5 mL of methanol, sulphuric acid, and toluene 2:1:1 (*v:v:v*), for at least 12 h at 50 °C and 160 rpm. Next, 3 mL of deionized water were added to obtain phase separation. The FAME were recovered with 3 mL of diethyl ether by shaking in vortex. A gas–liquid chromatography with flame ionization detection was performed using a YOUNG IN Crhomass 6500 GC System instrument equipped with a split/splitless injector set at 250 °C with a split ratio of 1:50, a flame ionization detector (FID) set at 260 °C, and a Zebron-Fame column (30 m). It was set to the following oven temperature program: Initial temperature of 100 °C, maintained for 2 min, increase of 10 °C/min to 140 °C, then a ramp of 3 °C/min to 190 °C, and a final ramp of 30 °C/min to 260 °C. At 250 °C, the carrier gas (hydrogen) flow rate was 1.2 mL/min. The results were described as a relative percentage of each fatty acid.

#### 2.2.4. Phenolic Compounds

Three distinct extracts were prepared by adding 1 g of dry sample (mangosteen lyophilized pericarp) to 30 mL of solvent, which contained (1) solution composed of 80% ethanol and 20% water for hydroethanolic extraction (MT80); (2) distilled water for aqueous extraction (MTW); and (3) ethanol (100%) for ethanolic extraction (MTE). For the extraction of anthocyanin compounds, the same solvents were acidified with citric acid (0.1%, 1 µM). The samples were extracted for 1 h under stirring at room temperature and filtrated (qualitative filter paper of 20–25 µm). After that, the residues were subsequently extracted a second time for 1 h under the same circumstances using additional 30 mL of the same solvent. The ethanol presented in the extracts was evaporated (at 40 °C) under vacuum condition, and aqueous phases of the hydroethanolic and aqueous extracts were lyophilized to produce dry extracts.

The freeze-dried extracts were resuspended (5 mg/mL) in of ethanol/water (20:80 *v/v*) and were analyzed using an HPLC-DAD-MSn, working under optimized conditions as in other studies [16,17]. The results were expressed as mg per g of extract (E) and mg per g of dw.

### 2.3. Assessment of the Bioactivities of Mangosteen Pericarp Extracts

#### 2.3.1. Antioxidant Potential

Two cell-based assays that measure the capacity to (1) prevent the generation of thiobarbituric acid reactive substances (TBARS) [15] and (2) postpone the oxidative hemolysis (OxHLIA) [18] were used to examine the antioxidant potential of the extracts (3.12–400 µg/mL). The TBARS results were defined as EC_50_ values (µg/mL), which represents the extract concentration that suppresses TBARS by 50%. The OxHLIA results were defined as IC_50_ values (µg/mL) for a Δ*t* of 60 min, which is the amount of extract needed to maintain 50% of the sheep erythrocyte population for 60 min. In both experiments, Trolox acted as a positive control.

#### 2.3.2. Anti-Inflammatory Potential

The ability of the extracts (6.25–400 g/mL) to prevent lipopolysaccharide-stimulated murine macrophage RAW 264.7 cells from producing nitric oxide (NO) was used to assess their anti-inflammatory potential [19]. A positive control was applied, which was Dexamethasone (50 μM). The results were represented as IC_50_ values (µg/mL), which correspond to the amount of the extract that causes 50% of the NO generation to be inhibited.

#### 2.3.3. Antiproliferative Potential

The evaluation of the antiproliferative potential of the extracts (6.25–400 µg/mL) was performed following the protocol for the sulforhodamine B (SRB) assay [20]. Four human tumor cell lines, namely NCI-H460 (non-small lung carcinoma cells); Caco-2 (colon adenocarcinoma cells); MCF-7 (breast carcinoma cells); and AGS (gastric adenocarcinoma cells), besides one non-tumor cell line obtained from African green monkey kidney (Vero), were used. The positive control employed was Ellipticine. The results were expressed as GI_50_ values (µg/mL), which correspond to the extract’s concentration required to inhibit 50% of cell proliferation.

#### 2.3.4. Antibacterial Potential

In order to evaluate the antibacterial potential of the extracts on pathogenic bacteria commonly causing nosocomial infections, six Gram-negative (*Escherichia coli*, *Klebsiella pneumoniae, Morganella morganni*, *Proteus mirabilis*, and *Pseudomonas aeruginosa*) and three Gram-positive (*Enterococcus faecalis*, *Listeria monocytogenes*, and Methicillin-resistant *Staphylococcus aureus* (MRSA)) bacteria were selected. Following a protocol established [21], the extracts were re-dissolved in water (20 mg/mL) and successive dilutions were carried out in a 96-well plate until 0.15 mg/mL. The minimum inhibitory concentrations (MIC), were determined by the rapid *p*-iodonitrotetrazolium chloride (INT) colorimetric assay. The minimum bactericidal concentrations (MBC) were determined by transferring a portion (10 µL) of each well that showed no color change to a blood agar medium (7% sheep blood) and incubated at 37 °C/24 h. MBC was determined as the lowest concentration capable of eradicating bacteria. Streptomycin and ampicillin were used as positive controls.

### 2.4. Statistical Analysis

The results of the analysis were expressed as mean value ± standard deviation, which were all carried out in triplicate. Statistical analyses were carried out using the R software (version 11). Student’s *t*-test was applied to detect statistical differences (*p* < 0.05) between two samples; for three samples, the analyses of variance (ANOVA) were used for detecting significant differences (*p* < 0.05) between them. The Tukey’s honest significant difference (HSD) test at the 5% of significance was applied to discriminate the samples.

## 3. Results and Discussion

### 3.1. Chemical Composition of Mangosteen Pericarp

#### 3.1.1. Organic Acids

The organic acid profile of mangosteen pericarp is presented in Table 1. Citric acid (56.72%) was the major compound detected in the sample, followed by quinic acid (17.99%). A low concentration of ascorbic acid was detected, whereas only traces of shikimic and fumaric acids were observed. Other studies reported different organic acid profiles for mangosteen pericarp samples. For instance, Mamat et al. [22] detected five organic acids by chromatography-mass spectrometry (GC-MS), namely malic, L-(+)-tartaric, citraconic, malonic and succinic acids when investigating mangosteen ripened pericarp. More recently, the same research team assessed the mangosteen metabolites throughout all ripening process (from green fruit (stage 0) to purple dark fruit (stage 6)) and detected traces of aspartic acid until stage 4 (brownish red), and the presence of ascorbic acid-2-glucoside, 2-butynedioic acid, and quinic acid was detected only in stage 2. Indeed, organic acid composition can be associated with the stage of fruit ripening [23]. In our investigation, only purple dark pericarps were analysed.

#### 3.1.2. Tocopherols

Tocopherol isomers present in the mangosteen pericarp were identified by HPLC-FD, and the results are shown in Table 1. In total, three tocopherol isomers were detected and quantified. β-tocopherol was the most abundant isomer, γ-tocopherol was detected in the lowest concentration, and α-tocopherol was detected in a median amount. Mangosteen pericarp showed a considerable amount of tocopherol isomers (9.9 mg/100 g dw).

Isabelle et al. [24] identified γ-, α-, and δ-tocopherol in samples of mangosteen flesh, α-tocopherol being the most abundant (5.74 mg/g fw) within a total tocopherol content of 9.9 mg/100 g dw. In another study concerning the *G. mangostana* pulp, the amount of vitamin *E* (α-tocopherol) quantified was 0.18 mg/100 g fw [25]. As expected, such composition of tocopherol is distinct from the one herein reported, which addresses only the mangosteen pericarp. Until the publication of this study, to the best of our knowledge, there was no previous report on the tocopherol profile of this part of the fruit.

#### 3.1.3. Fatty Acids

Mangosteen pericarp showed a low content of lipid (2.7 ± 0.6 g/100 g dw), and this lipid fraction was evaluated regarding its fatty acid composition, the results of which are presented in Table 1. Oleic acid was the most abundant fatty acid detected, followed by palmitic and linoleic acid, whereas stearic acid was the minor fatty acid constituent. In total, mangosteen pericarp showed 40.6% saturated, 34.1% monounsaturated, and 25.2% polyunsaturated fatty acids. The ratio between PUFA and SFA verified for the mangosteen shell of 0.62 can be considered appropriate for maintaining good health [26]. As far as we are aware, this is also the first research on the fatty acid profile of mangosteen pericarp. However, the fatty acid composition of the mangosteen seed has been described in the literature [27]. Similar to the pericarp, the mangosteen seed shows a higher amount of palmitic and oleic acid, besides linoleic acid in low concentrations [27].

#### 3.1.4. Phenolic Compound Composition

##### Non-Anthocyanin Compounds

The non-anthocyanin compounds present in the different extracts of mangosteen pericarp were assessed by LS-MS and the identification of the compounds was conducted considering their main characteristics in the mass spectral (mass fragmentation (MS²), maximum UV absorption (λmax)) and based on information obtained from the literature. The identification and quantification of phenolic compounds detected in the extracts are presented in Table 2 and Table 3, respectively.

Compound 1 ([M-H]^−^ at *m*/*z* 353) showed four fragment ions at MS² and comparing its mass spectrum with commercial standard, this compound was tentatively identified as a 5-*O*-caffeoylquinic acid. No data in the literature was discovered related to the presence of such compound in mangosteen fruit samples. Compounds 2, 4, and 11 ([M-H]^−^ at *m*/*z* 577) also showed the presence of ion fragment at *m*/*z* 289 after the loss of 288 Da, and based on its chromatographic characteristics and literature data, this compound was tentatively identified as procyanidin dimer [28]. Compounds 3, 6, and 10 ([M-H]^−^ at *m*/*z* 865) released three fragment ions at MS², corresponding to the successive breaking of bonds between monomers of epicatechin/catechin molecule (*m*/*z* 289). According to the mass spectrum and previous studies regarding mangosteen, these compounds have been identified as procyanidin trimer isomers [28]. The mass characteristics of compound 5 ([M-H]^−^ at *m*/*z* 289) were compared with commercial standards, and this compound was tentatively identified as a (+)-epicatechin. Regarding compounds 7 and 8 ([M-H]^−^ at *m*/*z* 1153), the analysis of MS² allowed the detection of four epicatechin/catechin molecules; however, these compounds were identified as procyanidin tetramer isomers, compounds previously identified in mangosteen pericarp by Zhou et al. [28]. Compound 9 ([M-H]^−^ at *m*/*z* 863) showed five fragment ions at MS²; according to the literature, this molecule has been discovered in mangosteen pericarp and identified as a procyanidin-A-like linkage [28]. Compound 12 ([M-H]^−^ at *m*/*z* 609) released a unique fragment ion MS² at *m*/*z* 301; the mass spectrum of this compound allowed its identification as a quercetin-3-*O*-rutinoside through DAD-MSn data. Compound 13 ([M-H]^−^ at *m*/*z* 449) showed two fragment ions at *m*/*z* 303 and *m*/*z* 285. Comparing its spectrum characteristics with those of standard compounds, this compound was identified as taxifolin-*O*-rhamnoside. These last two compounds have not been detected in mangosteen fruit before.

Most studies on the chemical composition of mangosteen fruit focus on xanthones, a restricted polyphenol class present in a small group of plants and fungi, as mangosteen fruit has been considered one of the major sources of such phytochemicals [9,31]. However, the present study focused on other classes of biocompounds. As a result, among non-anthocyanin compounds, condensed tannins were the most abundant polyphenols present in all extracts of mangosteen pericarp. In addition, one phenolic acid, two flavonoids, one flavonol and one flavanonol, were detected in this by-product.

According to Table 3, the hydroethanolic extract (MT80) showed the higher amount of phenolics among samples (MT80 > MTE > MTW). However, proanthocyanidins were the most abundant compound class present in all extracts, accounting for 95%, 94% and 90% of the total phenolic compounds non-anthocyanin in MT80, MTE, and MTW, respectively. 5-*O*-caffeoylquinic and traces of quercetin-3-*O*-rutinoside (Compound 12) were detected only in MT80, whereas taxifolin-*O*-rhamnoside (Compound 13) was detected in low concentration in all extracts, which, as far as we know, is an unprecedented information regarding the phenolic profile of mangosteen fruit. MTW showed the lowest total phenolic compound non-anthocyanin, and the concentration of each compound detected in it is also lower than the concentration present in the other extracts. Such result can be associated with the poor solubility of proanthocyanidins in water [32].

Only few works have described in detail the phenolic composition of the mangosteen pericarp. For example, Zarena and Sankar [33] identified the presence of thirteen phenolic acids in the fractionated and hydrolysed extract of mangosteen fruit shell. These authors concluded that most of the phenolic acids naturally present in this bioresidue are bound to glucoside molecules [33]. Zhou et al. [28] identified proanthocyanidins from purified extracts of mangosteen pericarp that showed high antioxidant activity by chemimal methods, namely ferric reduncing antioxidant power (FRAP), Trolox equivalent antioxidant capacity (TEAC), and DPPH radiacal scavenging capacity.

##### Anthocyanin Compounds

According to the anthocyanin composition data shown in Table 2, only two anthocyanin compounds were detected in the MT80 extract. The first one detected was compound 14 ([M]^+^ at *m*/*z* 611) that released MS² fragment at *m*/*z* 287 (−324 u, loss of two hexose units), suggesting the presence of a cyanidin-*O*-dihexoside. The same fragmentation behaviour was previously described in the identification of cyanidin-*O*-sophoroside in this part of mangosteen fruit [29,30]. The other anthocyanin compound (Compound 15 ([M]^+^ at *m*/*z* 435) was identified as delphinidin-*O*-pentoside due to the loss −132 u, which revealed the MS² fragmentation *m*/*z* 303, characteristic of an aglycone delphinidin. Following our knowledge, this is the first time that a delphinidin derivate is detected in the mangosteen fruit.

Zarena and Sankar [30] reported the identification of other two anthocyanins in mangosteen pericarp, namely pelargonidin-3-*O*-glucoside and cyanidin-3-*O*-glucoside. On the other hand, Yenrina et al. [34] did not detect such components in their mangosteen pericarp samples. Palapol et al. [29], which evaluated the anthocyanin composition of mangosteen fruit during repining, also reported the presence of cyanidin-3-*O*-glucoside in this bioresidue, besides other anthocyanins, such as cyanidin-glucoside-pentoxide and other three cyanidin derivates. In their studies, cyanidin-*O*-sophoroside was the most abundant anthocyanin compound detected, which corroborates our findings [29,30].

According to Table 3, the hydroethanolic extract (MT80) showed a 3.66 ± 0.02 mg/g E value equivalent to 1.062 ± 0.007 mg/g dw. This result is slightly lower than the one reported by Cheok et al. [35] when analyzing extracts obtained by conventional extraction with ethanol 70% (1.62 mg/g dw); on the other hand, the same authors registered the amount of 2.92 mg/g dw of anthocyanin for the extract produced via ultrasound-assisted extraction with methanol 70%. Another study performed by Muzykiewicz et al. [36] shows that anthocyanin extraction by ultrasound process from mangosteen epicarp is dependent on the solvent concentration, time of extraction, and initial condition of the pericarp (whether fresh or frozen (−20 °C)). According to the authors, the highest anthocyanin recovery yield (±24 mg cyanidin-3-*O*-glucoside/L) was obtained with extraction time of 60 min, ethanol 70% as solvent and fresh pericarp. Interestingly, the authors registered the lowest amounts of anthocyanin recovered when using the minimal and the maximum ethanol concentration (20% and 96%, respectively) [36], which is similar to what happened in our study, where no anthocyanin compounds were detected for minimal and maximum ethanol concentrations (Table 3). Therefore, selective methods and optimized conditions can improve the recovery of this specific class of color compounds present in mangosteen pericarp.

### 3.2. Bioactive Potentials of the Hydroethanolic and Aqueous Mangosteen Pericarp Extracts

#### 3.2.1. Antioxidant Potential

**Figure 2:**
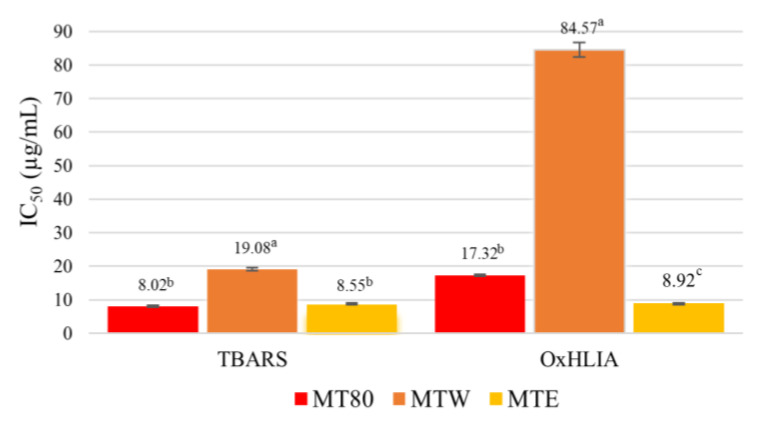
Antioxidant potential of the mangosteen pericarp extracts. Significant differences between samples in the same assay are indicated by different letters on the top of the bars (Tukey HSD test, *p* < 0.05).

The different extracts obtained from mangosteen pericarp were evaluated regarding their antioxidant potential. According to the results presented in Figure 2, all extracts have the capability to prevent lipid oxidation and oxidative hemolysis. However, the MT80 and MTE, which showed similar activities, were more efficient in inhibiting lipid oxidation than MTW, although no sample demonstrated a Trolox-like potential (IC_50_ value = 5.8 ± 0.6). Regarding the preservation of the blood erythrocytes, the MTE showed the best activity, being more protective than the positive control (Trolox, IC_50_ value = 19.6 ± 0.7 µg/mL), whereas MT80 had an antioxidant activity equivalent to that of the control, and a higher concentration of MTW was necessary to keep 50% of erythrocytes intact. In the study performed by Muzykiewicz et al. [36], extracts obtained by ultrasound showed better antioxidant activity when the solvent used had an ethanol concentration greater than 20%. Some studies suggested that the mangosteen pericarp has more antioxidant activity than the corresponding edible part, which has been correlated with its higher amount of phenolic compounds [12]. Among the phenolic compound classes, tannin and phenolic acid fractions of mangosteen have shown scavenging free radical capacity and anti-lipid peroxidation [28,33]. Some studies show that the tannin fraction has more free radical scavenging activity than the xanthone fraction. However, young fruits (rich in tannins, IC_50_ = 5.56 µg/mL) are more antioxidant than mature fruit (rich in xanthones, IC_50_ > 150 mg/mL) due to the change in phenolic composition occurring throughout ripening. In another study, xanthones isolated from mangosteen pericarp have shown less antioxidant activity than a crude extract of this by-product. In the study by Ngawhirunpat et al. [32], isolated α-mangostin showed lower antioxidant potential than isolated epicatechin and tannin. For instance, α-mangostin, the major compound of this class present in mangosteen, did not show free radical scavenging ability in DPPH assay (EC_50_ > 150 µg/mL) [2]. According to previous reports, phenolic compounds are mainly responsible for the antioxidant activity of mangosteen, especially ellagitannin derivatives [32]. In our study, MT80 had the highest concentration of phenolic compounds, which could justify its potent lipid oxidation inhibition. However, MTE was the most antioxidant extract in the OxHLIA system, what indicates that other classes of bioactive compounds, not identified in this study (such as xanthones) may also contribute for its antioxidant potential.

As demonstrated herein, the antioxidant potential of the extracts depends on the ethanol concentration (Figure 2). Several studies on diverse vegetal matrices have demonstrated that the antioxidant potentials of extracts obtained with the binary solvent water + ethanol tends to increase with the ethanol concentration between 60 and 80%, which is also correlated with the amounts of phenolic acids and flavanols recovered, as well as with the total phenolic content [37,38,39,40,41].

#### 3.2.2. Anti-Inflammatory Potential

Mangosteen pericarp extracts were evaluated regarding their ability to inhibit NO production on RAW 264.7 cells. Only MT80 and MTE had moderate anti-inflammatory potential, once their IC_50_ values, 85 ± 9 and 341 ± 2 μg/mL, respectively, were more than 5-fold higher than the concentration required for the positive control (Dexamethasone, IC_50_ value = 16 ± 1 μg/mL). On the other hand, the aqueous extract did not show inhibition of NO production at the highest concentration tested, which is likely related to the low concentration of bioactivity detected in this extract. Moreover, although the determination of xanthone compounds was not carried out in this study, it is known that this compound class has low solubility in water [11]. Hence, the combination of the factors cited above may justify the low anti-inflammatory potential verified for the MTW extract. Other studies have reported the potential of mangosteen extracts and their isolated compounds as anti-inflammatory agents. For instance, a low concentration (IC_50_ = 1 µg/mL) of an ethanolic extract obtained from exhaustive maceration of mangosteen pericarp was required to inhibit the NO production by RAW 264.7 cells [42]. In the same work, isolated xanthones, namely α- and γ-mangostin, showed IC_50_ values of 3.1 and 6.0 µM, respectively. Likewise, the proanthocyanidins present in the mangosteen pericarp showed the ability to bind LPS and neutralize its cytotoxicity [43]. Furthermore, the administration of silver nanoparticle biosynthesized with mangosteen pericarp extract to mice a dosage of 5 mg/mL/day for one week was able to inhibit the development of *Listeria*-induced infection [44]. The body of evidence mentioned above indicates that perhaps, after more specific studies, the pericarp of mangosteen and its isolated compounds may become a natural alternative to the traditional medicines used to control inflammation.

#### 3.2.3. Antiproliferative Potential

**Figure 3:**
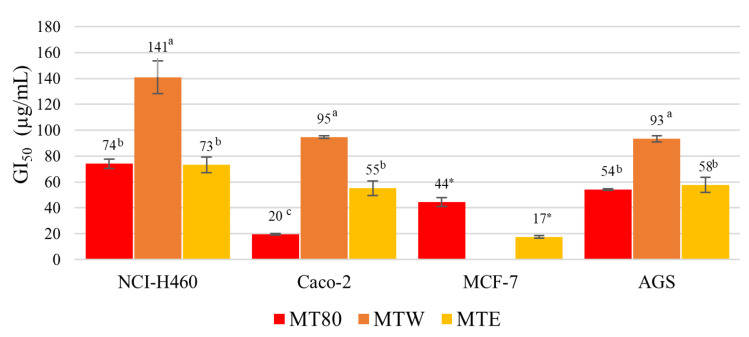
Antiproliferative potential of the mangosteen pericarp extracts. Significant differences between samples are indicated by different letters (Tukey HSD test, *p* < 0.05) or an asterisk (*) (Student’s *t*-test, *p* < 0.05) on the top of the bars.

Numerous studies have proven the anticancer properties of the xanthone extracts and isolated compounds from the mangosteen fruit. These compounds, mainly α-mangostin, have shown high antiproliferative activity on diverse tumor cell lines [10]. However, the present study focused on the determination of the antiproliferative activity of crude extracts poor in xanthone compounds. The results obtained are presented in Figure 3. All extracts showed cytotoxicity on NCI-H460, AGS and Caco-2 cell lines: for both lines, lower MT80 concentrations were required (GI_50_ values = 19–74 µg/mL), whereas higher MTW concentrations were needed (GI_50_ values = 93–141 µg/mL). MTE showed the highest antiproliferative activity on MCF-7 cells among samples, while MTW did not show antiproliferative action at the highest concentration tested. According to the literature, compounds from mangosteen fruit belonging to the xanthone class have shown anticancer proprieties against several malignant cell lines [45,46,47]. Moreover, mangosteen pericarp extracts displayed inhibitory activities against hepatocellular carcinoma in an animal model [47]. The action against the proliferation of HeLa cells was determined in a crude hydroethanolic extract (GI_50_ value of 18.087 μg/mL) of mangosteen pericarp, whereas the isolated α-mangosteen was highly cytotoxic on this cancer cell line (GI_50_ values of 6.5 μg/mL) [48]. MTW showed considerable antiproliferative potential against Caco-2 and AGS tumor-cell lines. Such bioactivity can be related, inter alia, to the presence of proanthocyanidin compounds detected in this extract [49].

Regarding the antiproliferative potential of our extracts on the Vero cell line, the MT80 and MTW extracts did not show toxicity in the highest concentration tested. Although the MTE extract was harmful to the proliferation of VERO cells, it only happened in a concentration (GI_50_ value of 76 ± 7 µg/mL) higher than the one required to inhibit the proliferation of tumor cell lines (GI_50_ values 17–73 µg/mL). Similar results were reported in the study performed by Ngawhirunpat et al. [32] using water, methanol and hexane as solvents. According to the authors, the first extract did not show toxicity in human keratinocyte cells (HaCat) in the maximal level tested, whereas the other extracts and isolated compounds were harmful to cell viability (GI_50_ values were 72, 30 and 2.5 μg/mL, respectively). Their aqueous extract did not show α-mangostin in its composition, while this compound was quantified in high amounts in their other extracts (15.5 and 18.7% (*w*/*w*), in methanol and hexane extracts, respectively).

Finally, it is worth noting that in the balance between the antiproliferative potential on tumor and non-tumor cell lines tested, the hydroethanolic extract could be considered safe for the development of anticancer drugs. In addition, previous in vitro studies have also verified the toxicity of different extracts obtained from mangosteen pericarp and their isolated compounds, namely xanthones and proanthocyanidins, towards diverse non-cancerous human cell lines.

#### 3.2.4. Antibacterial Potential

The minimal inhibitory concentration (MIC) of the extracts required for each bacteria tested is shown in Table 4. The pericarp samples exhibited antibacterial potential against all the bacteria tested, except against *P. aeruginosa* to which no inhibition was observed in the highest concentration of extract tested (20 mg/mL). However, no extract showed bactericidal effect at the highest concentration tested. Lower extract concentrations were required to inhibit the proliferation of Gram-positive bacteria (0.625–1.25 mg/mL) rather than of Gram-negative bacteria (2.5–10 mg/mL). The same tendency was reported by Taokaew et al. [50] when investigating the antibacterial potential of a multifunctional cellulosic nanofiber enhanced with mangosteen pericarp extract. They believe that this activity is related to the ability of α-mangostin to diffuse through the cell membrane of Gram-positive bacteria. In another study, aqueous extracts obtained from mangosteen by-products did not show antibacterial potential against Gram-positive bacteria, as their extracts had a low concentration of bioactive compounds, namely tartaric acid and flavonols, and likely less ability to cause damage to the cell membrane of the Gram-positive bacteria [51]. Furthermore, according to the extensive review performed by Lima et al. [52], fruit extracts in methanol and ethanol are more effective in inhibiting pathogenic bacteria than fruit extracts obtained with water as solvent.

According to the literature, xanthones from mangosteen hold high antibacterial activity towards several bacterial strains, such as *Propionibacterium acnes*, *Staphylococcus epidermidis*, and *Streptococcus mutans* [2,53]. On the other hand, proanthocyanidins from mangosteen have been shown to contribute to the inhibition of *L. monocytogenes* growth [54]. Regarding potential applications, the antibacterial activity of mangosteen pericarp extract and its derivatives have been explored for the development of dye cotton with properties against *S. aureus* and *E. coli* [55], self-nanoemulsifying drug delivery with action against *Staphylococcus epidermidis* [56], and for the production of medical glove films with antimicrobial activity [57].

Compared to other fruit by-products, mangosteen pericarp has greater antibacterial potential against *E. coli*, *S. aureus*, and *L. monocytogenes* than *Punica granatum* L. pericarp (MIC= 50–60 mg/mL), *Sambucus nigra* L. peels and seeds (MIC = 7.81–15.63 mg/mL), and Prunus domestica L. peels (MIC = 7.81–15.63 mg/mL) [52]. As a result, it might be a fascinating source of antimicrobial substances.

## 4. Conclusions

Mangosteen is an exotic fruit with cultural and economic relevance in Asia countries. Its industrial exploitation generates huge amount of by-products, as the inedible pericarp can constitute more than 50% of the whole fruit. Some researchers have focused on the xanthone composition of the mangosteen residue; however, our study showed that other interesting bioactive compounds can be recovered from this biomass. Among them, proanthocyanidins are the most abundant, the successful recovery of which depends on solvent extraction. In the conditions tested in this work, the hydroethanolic solvent (80%) was the most efficient for proanthocyanidin recovery, as anthocyanin compounds and a quercetin derivative were only detected in this extract (MT80). Moreover, the MT80 extract showed good bioactivity, such as high antioxidant, antiproliferative, anti-inflammatory and antibacterial potential. However, it is worth nothing that a good bioactive potential was also verified for the ethanol extract, although this extract showed some cytotoxic at a low concentration. Taken together, our results support the suggestion that the ripe mangosteen pericarp is a source of proanthocyanidins with promising biological activities, with potential to be upcycled into multifunctional ingredients for the food and pharmacological industries.

## Figures and Tables

**Table 1:** The *G. mangostana* pericarp’s composition in terms of organic acids, tocopherols, and fatty acids.

**Organic Acids**	**Content (g/100 g)**
Oxalic acid	0.208 ± 0.001
Quinic acid	0.241 ± 0.002
Malic acid	0.11 ± 0.01
Ascorbic acid	0.017 ± 0.001
Shikimic acid	tr
Citric acid	0.76 ± 0.04
Fumaric acid	tr
Total organic acids	1.34 ± 0.04
**Tocopherols**	**Content (mg/100 g)**
α-tocopherol	1.94 ± 0.05
β-tocopherol	7.3 ± 0.2
γ-tocopherol	0.69 ± 0.05
Total tocopherols	9.9 ± 0.1
**Fatty Acids**	**Relative %**
Palmitic acid (C16:0)	28.64 ± 0.04
Stearic acid (C18:0)	12.0 ± 0.3
Oleic acid (C18:1n9c)	34.2 ± 0.3
Linoleic acid (C18:2n6c)	25.21 ± 0.04
SFA	40.6 ± 0.3
MUFA	34.1 ± 0.3
PUFA	25.2 ± 0.04
PUFA/SFA	0.62 ± 0.01

tr—traces; dw—dry weight; oxalic acid (*y* = 1*E* + 7*x* + 231,891; R² = 0.9999); malic acid (*y* = 950,041*x* + 6255.6; R² = 0.9999); shikinic acid (*y* = 5*E* + 7*x* + 109,778; R² = 0.9999); citric acid (*y* = 1*E* + 6*x* + 10,277; R² = 0.9997); fumaric acid (*y* = 1*E* + 8*x* + 614,399; R² = 0.9986). SFA—saturated fatty acids; MUFA—monounsaturated fatty acids; PUFA—polyunsaturated fatty acids.

**Table 2:** Phenolic compounds detected in different extracts of the mangosteen pericarp extracts.

Compound	Rt (min)	λ_max_ (nm)	[M-H]^−/+^ (*m*/*z*)	MS² (*m*/*z*)	Tentative Identification	Reference
Non-anthocyanin compounds						
1	6.99	326	353	191(100), 179(10), 173(5), 135(5)	5-*O*-Caffeoylquinic	DAD-MSn
2	6.96	280	577	451(29), 425(100), 407(30), 289(15)	B-Type (epi)catechin dimer	[28]
3	7.26	280	865	577(85), 289(5), 287(30)	Procyanidin trimer	[28]
4	7.71	280	577	451(20), 425(100), 289(12)	Procyanidin dimer	[28]
5	9.43	280	289	245(100), 205(44)	(+)-Epicatechin	DAD-MSn
6	10.79	280	865	577(85), 289(5), 287(25)	Procyanidin trimer	[28]
7	11.98	280	1153	865(100), 577(29), 289(34)	Procyanidin tetramer	[28]
8	12.61	280	1153	865(34), 577(86), 289(21)	Procyanidin tetramer	[28]
9	13.31	280	863	711(100), 573(20), 451(34), 411(19), 289(5)	Procyanidin with A-type linkage	[28]
10	14.51	280	865	577(85), 289(5), 287(34)	Procyanidin trimer	[28]
11	15.32	280	577	451(31), 425(100), 407(34), 289(10)	B-Type (epi)catechin dimer	[28]
12	17.76	326	609	301(100)	Quercetin-3-*O*-rutinoside	DAD-MSn
13	18.91	295	449	303(100),285(25)	Taxifolin-*O*-rhamnoside	DAD-MSn
Anthocyanin compounds						
14	13.27	515	611	287(100)	Cyanidin-*O*-sophoroside	[29,30]
15	16.11	515	435	303(100)	Delphinidin-*O*-pentoside	DAD-MSn

Rt—retention time; λ_max_—wavelength of maximum absorption; [M-H]—pseudomolecular ion; *m*/*z*—charge–mass ratio.

**Table 3:** Quantification of phenolic compounds detected in different extracts of the mangosteen pericarp.

Compound	mg/g Extract			mg/g Dry Pericarp		
	MT80	MTW	MTE	MT80	MTW	MTE
1	1.30 ± 0.08	nd	nd	0.40 ± 0.02	nd	nd
2	nd	1.01 ± 0.03 *	4.16 ± 0.03 *	nd	0.222 ± 0.007 *	0.86 ± 0.01 *
3	3.66 ± 0.09	nd	nd	1.13 ± 0.03	nd	nd
4	11.2 ± 0.2	nd	nd	3.47 ± 0.05	nd	nd
5	12.3 ± 0.3 ^a^	0.58 ± 0.01 ^c^	3.7 ± 0.1 ^b^	3.78 ± 0.10 ^A^	0.127 ± 0.002 ^C^	0.77 ± 0.02 ^B^
6	9.6 ± 0.3 ^a^	0.43 ± 0.04 ^c^	2.0 ± 0.1 ^b^	2.98 ± 0.09 ^A^	0.094 ± 0.008 ^C^	0.42 ± 0.03 ^B^
7	8.4 ± 0.04 ^a^	0.37 ± 0.01 ^c^	2.23 ± 0.05 ^b^	2.59 ± 0.01 ^A^	0.082 ± 0.001 ^C^	0.46 ± 0.01 ^B^
8	nd	0.472 ± 0.005 *	2.02 ± 0.09 *	nd	nd	0.42 ± 0.02
9	3.4 ± 0.2	nd	nd	1.04 ± 0.06	nd	nd
10	2.96 ± 0.01 ^a^	0.30 ± 0.01 ^c^	2.22 ± 0.03 ^b^	0.913 ± 0.002 ^A^	0.104 ± 0.001 ^C^	0.46 ± 0.01 ^B^
11	nd	0.433 ± 0.002 ^b^	1.82 ± 0.01 ^a^	nd	0.065 ± 0.001 *	0.3756 ± 0.0001 *
12	tr	nd	nd	tr	nd	nd
13	1.02 ± 0.05 ^a^	0.41 ± 0.01 ^b^	1.05 ± 0.04 ^a^	0.32 ± 0.02 ^A^	0.095 ± 0.001 ^C^	0.22 ± 0.01 ^B^
14	2.41 ± 0.03	nd	nd	0.699 ± 0.009	nd	nd
15	1.25 ± 0.01	nd	nd	0.363 ± 0.002	nd	nd
TPC-NA	54 ± 1 ^a^	4.011 ± 0.005 ^b^	19.79 ± 0.08 ^c^	16.6 ± 0.4 ^A^	0.879 ± 0.001 ^C^	3.99 ± 0.04 ^B^
TA	3.66 ± 0.02	nd	nd	1.062 ± 0.007	nd	nd

Tr—traces; nd—not detected; TPC-NA—total phenolic compound non-anthocyanin; TA—total anthocyanin. Standard compounds used for quantification: chlorogenic acid (*y* = 168,823*x* − 161,172, R² = 0.9999, limit of detection (LOD) = 0.83 µg/mL, limit of quantification (LOQ) = 2.50 µg/mL, for compound 1), catechin (*y* = 84,950*x* − 23,200, R² = 1, LOD = 0.44 µg/mL, LOQ = 1.33 µg/mL, for compounds 2–10), quercetin-3-*O*-rutinoside (*y* = 13,343*x* +76,751, R² = 0.9998, LOD = 14.71 µg/mL, LOQ = 44.59 µg/mL, for compound 12), taxifolin (*y* = 203,766*x* − 208,383, R² = 1, LOD = 0.67 µg/mL, LOQ = 2.02 µg/mL, for compound 13), and cyanidin-3-*O*-glucoside (*y* = 134,578*x* − 3E + 06, R² = 0.9986, LOD = 9.94 µg/mL, LOQ = 30.13 µg/mL, for compounds 14–15). Different letters or an asterisk (*) on the same line means significant difference between samples (*p* < 0.05) determined by the Tukey HSD test or Student’s *t*-test, respectively.

**Table 4:** Antibacterial potential of the mangosteen pericarp extracts.

	MT80	MTW	MTE	Ampicillin	
	MIC	MIC	MIC	MIC	MBC
Gram-negative bacteria					
*Escherichia coli*	2.5	10	10	<0.15	<0.15
*Klebsiella pneumoniae*	10	10	>20	10	20
*Morganella morganii*	10	10	20	20	>20
*Proteus mirabilis*	10	10	>20	<0.15	<0.15
*Pseudomonas aeruginosa*	>20	>20	20	>20	>20
Gram-positive bacteria					
*Enterococcus faecalis*	0.625	5	2.5	<0.15	<0.15
*Listeria monocytogenes*	1.25	2.5	2.5	<0.15	<0.15
Methicillin-resistant *Staphylococcus aureus*	1.25	5	1.25	<0.15	<0.15

## Data Availability

Data is contained within the article.
